# Global trends and research hotspots in autophagy and tumor drug resistance: a bibliometric analysis

**DOI:** 10.1007/s12672-025-02379-5

**Published:** 2025-05-12

**Authors:** Long Zhao, Jiao Zeng, Junfeng Wen, Zhaoyang Li, Jianguo Xu, Jinxiang Wang, Xiaoping Tang, Lingmi Hou

**Affiliations:** 1 Department of Neurosurgery, Affiliated Hospital of North Sichuan Medical College, No. 1 South Maoyuan Road, Shunqing District, Nanchong, 637000 Sichuan People’s Republic of China; 2 Department of Clinical Medicine, North Sichuan Medical College, Nanchong, 637000 People’s Republic of China; 3 Department of Neurosurgery, West China Hospital, Sichuan University, Chengdu, 610041 People’s Republic of China; 4 Department of Urology, Kidney and Urology Center, Pelvic Floor Disorders Center, The Seventh Affiliated Hospital, Sun Yat-Sen University, Shenzhen, 518107 Guangdong People’s Republic of China; 5 Department of Breast Surgery, Sichuan Clinical Research Center for Cancer, Sichuan Cancer Hospital & Institute, Sichuan Cancer Center, Affiliated Cancer Hospital of University of Electronic Science and Technology of China, No. 55, Section 4, Renmin South Road, Wuhou District, Chengdu, 610041 Sichuan People’s Republic of China

**Keywords:** Autophagy, Tumor drug resistance, Chemotherapy resistance, Bibliometric analysis

## Abstract

Autophagy plays a crucial role in tumor drug resistance by enabling cancer cells to survive under stress conditions, including chemotherapy. It helps tumor cells maintain homeostasis, resist cell death, and contribute to therapy failure. This study analyzed the literature related to autophagy and tumor drug resistance based on the Web of Science Core Collection (WoSCC) database. The results revealed that there are 9284 relevant articles published to date, covering 103 countries and regions, with contributions from 5964 institutions and 37,240 researchers. The annual number of publications has steadily increased since 2004, especially after 2019, indicating the growing importance of autophagy in tumor drug resistance research. China leads globally in terms of publication output, accounting for nearly 50% of the total publications. Additionally, international collaboration and cross-country research have become increasingly prominent, particularly collaborations between China and countries like South Korea and Japan. Journal analysis showed that the International Journal of Molecular Sciences and Oncotarget are the most productive journals, while Autophagy stands out with a higher impact factor. Author, citation, and keyword analyses revealed research hotspots and future trends in the field of autophagy and tumor drug resistance, including chemotherapy resistance, cell death mechanisms, and immunotherapy. This study provides a systematic academic perspective for future research in the field of autophagy and tumor drug resistance and emphasizes the importance of strengthening international cooperation.

## Introduction

Cancer is one of the leading causes of death globally, and its development is influenced by multiple factors [1, 2]. Epidemiological studies show that the incidence of cancer continues to rise worldwide, especially in developed countries and some developing countries [3, 4]. Lung cancer, breast cancer, colorectal cancer, liver cancer, and gastric cancer are the most common types of cancer. The occurrence of cancer is closely related to genetic factors, as well as environmental factors, lifestyle choices (such as smoking, poor diet, lack of physical activity), and infections (such as HPV infection with cervical cancer, hepatitis B virus with liver cancer) [5, 6]. The epidemiological characteristics of cancer vary based on factors such as region, gender, age, and ethnicity [7, 8]. Current cancer research focuses on early diagnosis, targeted therapy, immunotherapy, and precision medicine. With advancements in genomics and molecular biology, significant progress has been made in understanding the molecular mechanisms of cancer, yet many challenges remain [9, 10]. The heterogeneity of cancer cells leads to varied responses to treatment, and many patients develop resistance to existing treatments such as chemotherapy and targeted therapy [11, 12]. Moreover, immune evasion mechanisms and angiogenesis within the tumor microenvironment significantly reduce treatment effectiveness. The role of immune evasion and alterations in tumor-associated immune cells continue to be key areas of cancer research [13–15]. Epidemiological studies provide us with patterns of cancer incidence and risk factors, revealing trends in different cancer types. For example, the high incidence of certain cancers is closely linked to regional environmental pollution, dietary habits, and lifestyle, while others are related to specific viral infections such as hepatitis B or HPV. In low-income countries and regions, early diagnosis and treatment of cancer face significant challenges due to limited medical resources [16]. Therefore, improving early cancer screening, promoting healthy diets and lifestyles, and encouraging vaccination have become effective strategies for controlling cancer prevalence. Overall, despite significant progress in cancer research in terms of underlying mechanisms, early diagnosis, and treatment, many key issues remain unresolved, such as immune evasion, cancer heterogeneity, and treatment resistance. Future research needs to further explore the molecular mechanisms of cancer to provide better theoretical foundations for personalized treatment and precision medicine.

The lysosomal pathway is involved in autophagy, a cellular process that degrades and recycles damaged or unneeded cellular components. Among its many important functions are stress response, energy balance regulation, and cellular homeostasis maintenance [17, 18]. Autophagosomes are double-membraned vesicles that integrate with lysosomes to degrade and recycle cytoplasmic components, such as damaged organelles and proteins [19, 20]. Nutrient scarcity, hypoxia, and oxidative stress are among the triggers that might initiate autophagy. The relationship between autophagy and tumor resistance is complex. In the context of cancer, autophagy can have both tumor-suppressing and tumor-promoting effects [21–23]. On one hand, it helps tumor cells survive stressors such as chemotherapy or radiotherapy by degrading damaged cellular components, thereby promoting cell survival. This adaptive mechanism enables cancer cells to withstand unfavorable conditions and evade cell death, contributing to therapeutic resistance. On the other hand, autophagy can inhibit tumor initiation and progression by preventing the accumulation of damaged proteins and organelles that could otherwise drive malignancy. In the context of drug resistance, autophagy has been shown to be a crucial mediator of resistance to various chemotherapy and targeted therapies [24–26]. When cancer cells are exposed to chemotherapeutic agents, autophagy is often upregulated as a survival mechanism, allowing the cells to resist drug-induced cell death. Inhibiting autophagy has emerged as a potential therapeutic strategy to overcome drug resistance in tumors. However, the dual nature of autophagy in cancer complicates its targeting, as autophagy inhibition could promote tumorigenesis in some contexts while enhancing the effectiveness of cancer treatments in others [27–29]. Therefore, understanding the precise role of autophagy in cancer and its impact on drug resistance is critical for developing more effective therapeutic strategies.

Bibliometric analysis is a quantitative research method used to evaluate and analyze scientific literature through statistical and mathematical techniques [30–32]. It examines patterns, trends, and relationships in scholarly publications, including articles, books, conference papers, and patents. Key aspects of bibliometric analysis include citation analysis, where the number of citations is used to measure the impact of research; co-authorship analysis, which reveals collaboration patterns between authors; and keyword analysis, which identifies emerging research trends and popular topics [33, 34]. Additionally, bibliometric analysis evaluates journal impact factors, which reflect the influence of a journal in a particular field. Other techniques like bibliographic coupling and co-citation analysis help uncover thematic connections between publications [35–37]. Bibliometric software tools, such as VOSviewer and CiteSpace, are extensively utilized to visualize citation networks, identify key themes, and highlight influential papers. These tools are invaluable for assessing research productivity, identifying leading authors and institutions, mapping research domains, and informing funding decisions. Additionally, they help uncover knowledge gaps and track the evolution of specific research areas over time.

Several databases were searched for literature pertaining to autophagy and drug resistance, and then analyzed using bibliometric methods. Our goal was to compile a comprehensive overview of the most recent and influential studies on autophagy and drug resistance that have been conducted worldwide between 2004 and 2024.

## Methods and materials

### Data collection and search strategy

**Fig. 1:**
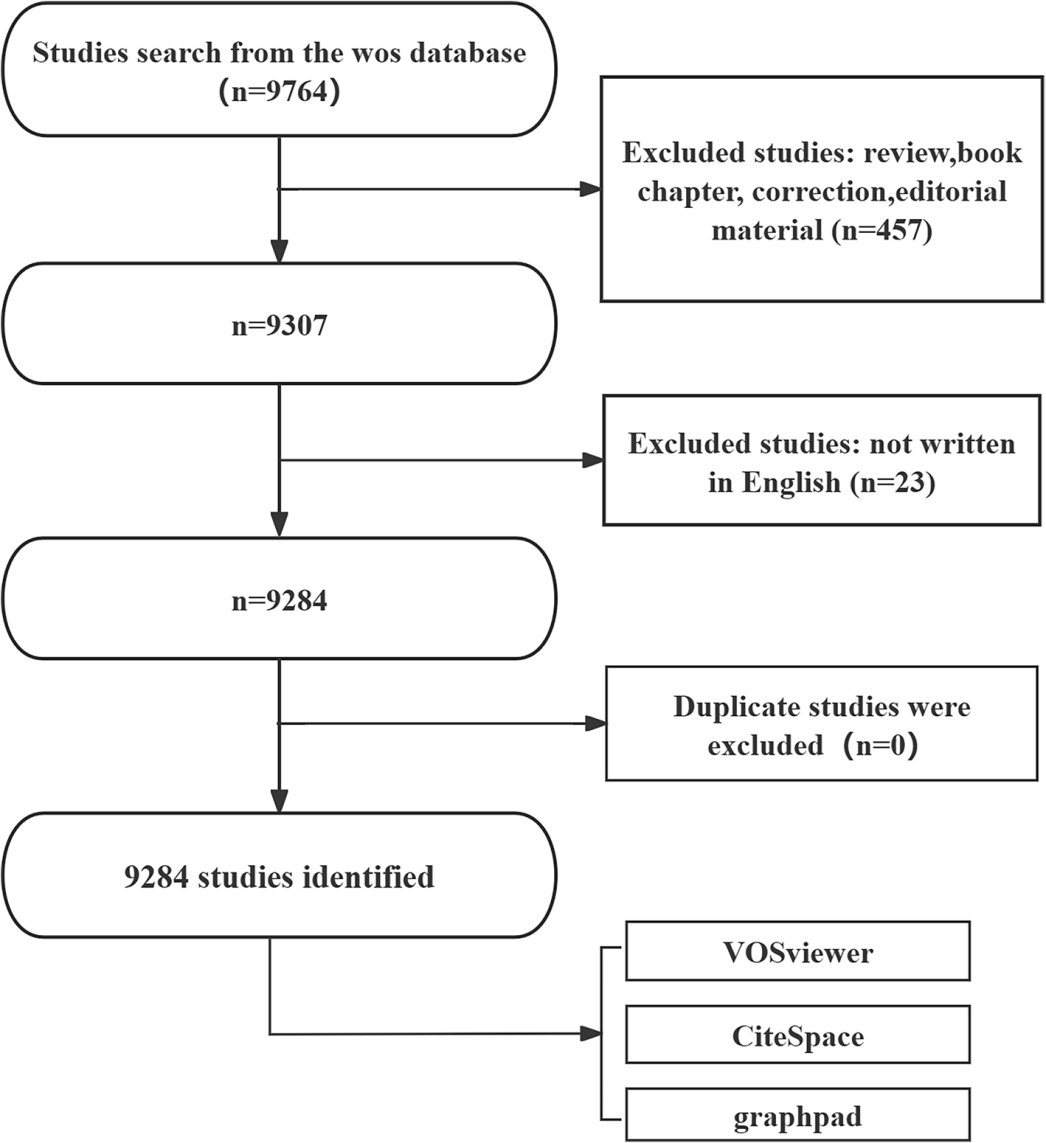
Flowchart illustrating the step-by-step process of the literature search, detailing the selection criteria, databases used, and methodology for including relevant studies

The Web of Science Core Collection (WoSCC) database is renowned for its superior accuracy in categorizing literature types compared to other databases, making it the preferred choice for bibliometric analysis. Consequently, we selected this database for our research. On November 19, 2024, we conducted a comprehensive search in WoSCC for all articles related to autophagy and tumor drug resistance using the following search formula: ((TS = (resistan*)) OR TS = (“drug resistan*”)) OR TS = (“drug-resistan*”) AND (((((((((TS = (Autophagy)) OR TS = (Autophagocytosis)) OR TS = (“Autophagy, Cellular”)) OR TS = (“Cellular Autophagy”)) OR TS = (Lipophagy)) OR TS = (Ribophagy)) OR TS = (Reticulophagy)) OR TS = (“ER-Phagy”)) OR TS = (“ER Phagy”)) OR TS = (“Nucleophagy”) AND TS = (“Tumor”) OR TS = (“Neoplasm”) OR TS = (“Tumors”) OR TS = (“Neoplasia”) OR TS = (“Neoplasias”) OR TS = (“Cancer”) OR TS = (“Cancers”) OR TS = (“Malignant Neoplasm”) OR TS = (“Malignancy”) OR TS = (“Malignancies”) OR TS = (“Malignant Neoplasms”) OR TS = (“Neoplasm, Malignant”) OR TS = (“Neoplasms, Malignant”) OR TS = (“Benign Neoplasms”) OR TS = (“Benign Neoplasm”) OR TS = (“Neoplasms, Benign”) OR TS = (“Neoplasm, Benign”). Our search resulted in 9284 relevant papers (Fig. 1).

### Inclusion and exclusion criteria with data analysis tools

The selection of literature for this study was based on the following inclusion criteria: (1) full-text publications related to autophagy in tumor drug resistance; and (2) articles and review manuscripts written in English. The exclusion criteria were: (1) publications unrelated to autophagy in tumor drug resistance; and (2) documents that fall within the categories of conference abstracts, news, and briefs. The chosen papers were saved as plain text. Annual publishing patterns, national publication trends, and proportions were analyzed and shown using GraphPad Prism v8.0.2. The data was analyzed and scientific knowledge maps were shown using VOSviewer (version1.6.18) and CiteSpace (version 6.2.4R, 64-bit advanced edition).

### Tools for bibliometric analysis and visualization

A free Java-based program called VOSviewer (version 1.6.18) was created by Waltman et al. in 2009 for the purpose of analyzing big bibliometric datasets and displaying the findings in a map format. Professor Chaomei Chen created CiteSpace (version 6.2.4R) to help researchers see the big picture of their field’s work by creating maps of co-citations. The goal behind this program is to use it as a springboard to test out different approaches and see what works. Users may anticipate future advancements in the subject and have a better grasp of knowledge domains, research frontiers, and trends with its help.

## Results

### Analysis of annual publications

**Fig. 2:**
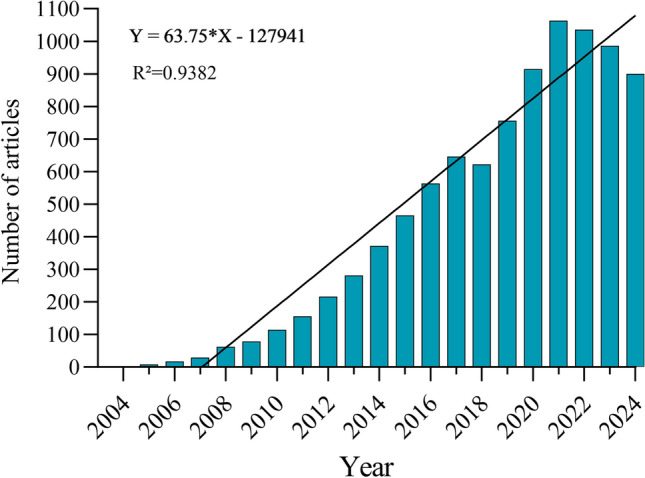
Annual volume of publications, showing the trend of published articles over a specific period, highlighting any noticeable increases or declines in research activity

Among the 9284 papers pertaining to autophagy and tumor treatment resistance included in the WoSCC database are 2707 reviews and 7077 articles. This field’s research is really worldwide, as these articles cover 103 nations and regions, include 5964 institutions, and are written by 37,240 researchers. The number of yearly releases has been rising steadily since 2004, with three separate periods. Reflecting the early phases of study in this domain, the development was sluggish from 2004 to 2009, with less than 100 publications produced each year. The increasing interest in autophagy’s function in tumor treatment resistance led to a steady increase in the number of publications from 2010 to 2018. The number of publications reached a peak in 2021 after 2019 (Fig. 2). The rapid advancement in this field can be attributed to the interplay of research technology, the growing recognition of autophagy’s role in cancer treatment resistance, financial support, and collaborative efforts. This trend underscores the critical importance of autophagy in cancer research and its potential to overcome resistance to tumor treatments.

### Global trends and institutional insights

**Fig. 3:**
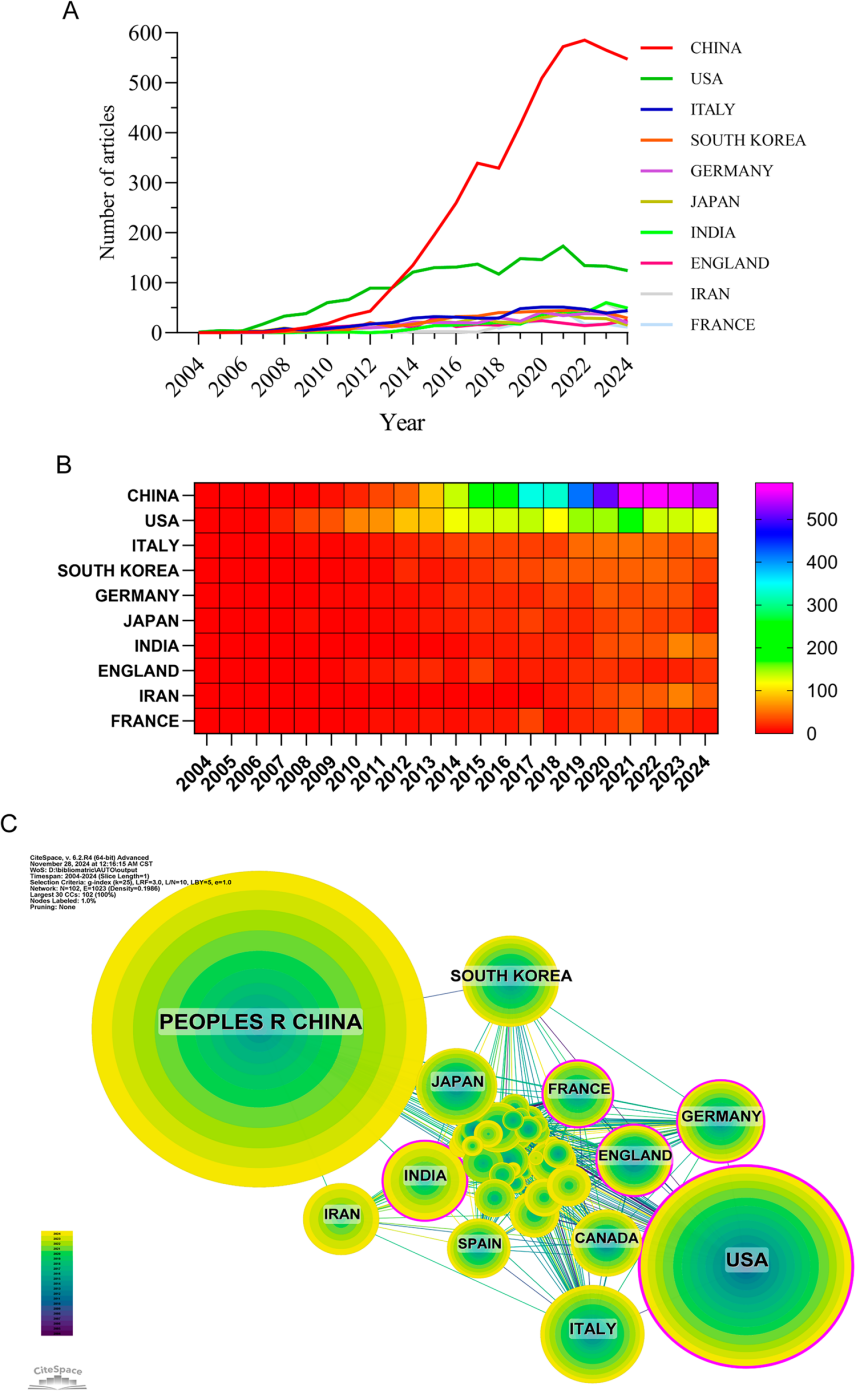
**A** Line graph depicting the number of national publications per year, offering a visual representation of publication trends across different years. **B** Heatmap representing the geographical distribution of national publications, allowing for a quick comparison of publication activity in different countries or regions. **C** China has strong research ties with South Korea, Japan, Iran, and India

**Fig. 4:**
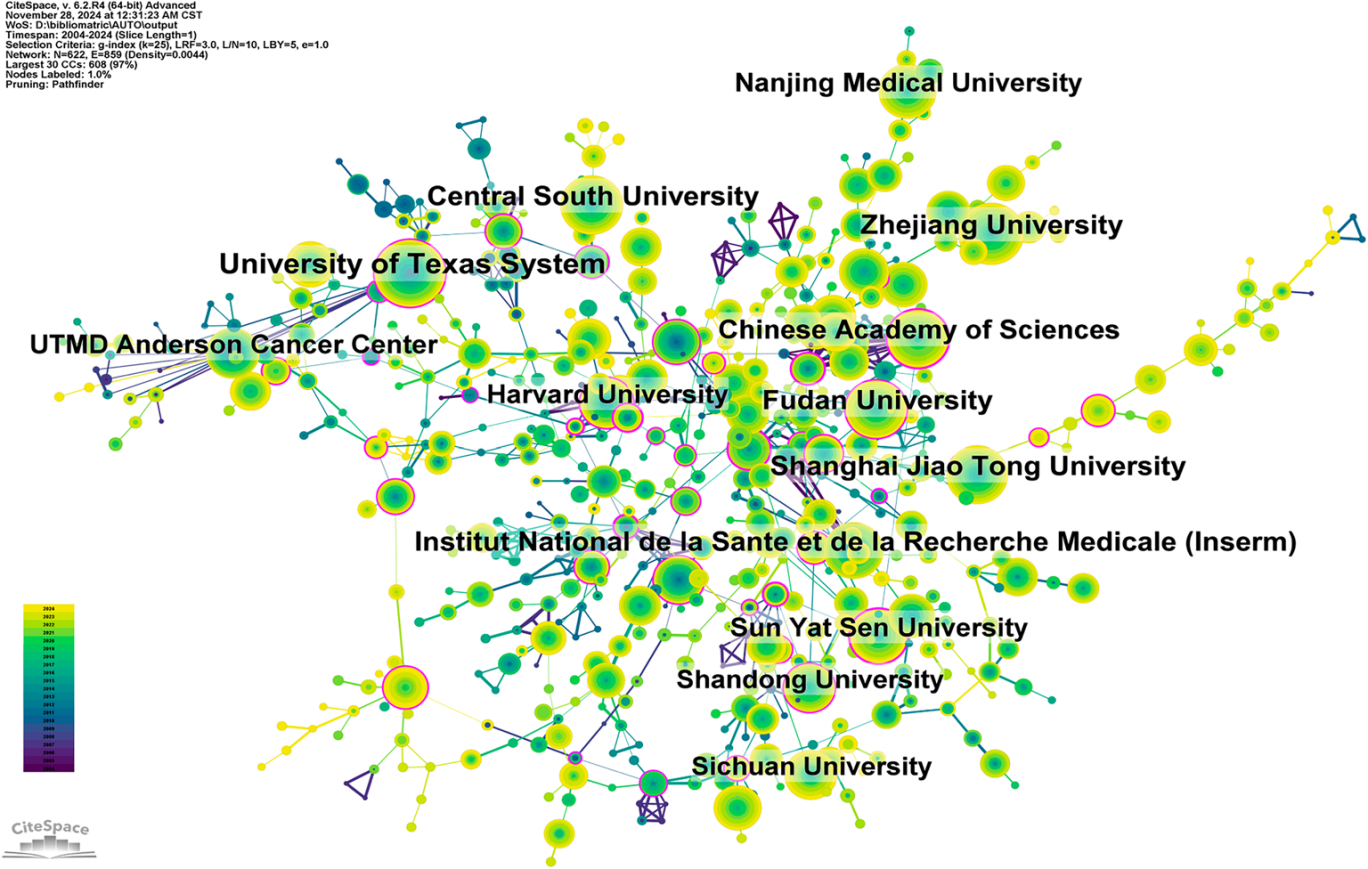
Network of institutional cooperation, visualizing the collaborative relationships between various institutions based on co-authored publications, emphasizing major research hubs

There have been studies in 103 different nations and areas looking into autophagy’s potential role in tumor treatment resistance. For the last 10 years, the top ten nations’ yearly publishing output was shown in Fig. 3A and B. Among these nations, China, the US, Italy, South Korea, and Germany rank first. It is worth mentioning that China has a far larger share of publications (49.99%) compared to other countries. With 147,486 citations (Table 1), China is far and by the most cited country or area in the top ten countries or regions by publication volume. The average quality of its publications is significantly lower, nevertheless, as its citation-to-publication ratio (31.78) ranks eighth. With a citation-to-publication ratio of 67.08, the United States is among the highest countries in terms of publication output (1894 articles) and citations (127,047). Figure 3C showed that China has strong research ties with South Korea, Japan, Iran, and India, whereas the US has strong ties with Canada, Germany, and the UK. Both the number of publications and the number of citations indicate that China is at the forefront of this subject. Systematic research on autophagy and tumor treatment resistance has been published by 5964 institutes. Table 2 and Fig. 4 showed that seven Chinese universities, two American institutions, and one French institution make up the top ten in terms of publishing volume. With 257 publications and 21,784 citations (94.76 citations per paper), the University of Texas System is in first place for production. Second place goes to Zhejiang University with 197 papers (7368 citations/paper = 37.40), third to Central South University with 190 papers (9373 citations/paper = 49.33), and last to Inserm with 186 papers (11,072 citations/paper = 59.53). When looking at domestic institutions more closely, it becomes clear that they mostly work with other domestic institutions. Hence, we supported more worldwide cooperation to remove academic obstacles and boost research synergy.

**Table 1:** Table of country published literature

Rank	Country/region	Article counts	Centrality	Percentage (%)	Citation	Citation per publication
1	China	4641	0.05	49.99	14,7486	31.78
2	USA	1894	0.21	20.40	12,7047	67.08
3	Italy	504	0.08	5.43	21,268	42.20
4	South Korea	441	0.01	4.75	15,100	34.24
5	Germany	347	0.16	3.74	16,578	47.78
6	Japan	323	0.07	3.48	14,210	43.99
7	India	317	0.13	3.41	8458	26.68
8	England	269	0.19	2.90	19,900	73.98
9	Iran	253	0.05	2.73	6413	25.35
10	France	251	0.11	2.70	14,730	58.69

**Table 2:** Table of institutional published literature

Rank	Institution	Country	Number of studies	Total citations	Average citation
1	University of Texas System	USA	257	21,784	84.76
2	Zhejiang University	China	197	7368	37.40
3	Central South University	China	190	9373	49.33
4	Institut National de la Sante et de la Recherche Medicale (Inserm)	France	186	11,072	59.53
5	Shanghai Jiao Tong University	China	184	7229	39.29
6	Fudan University	China	182	6618	36.36
7	Chinese Academy of Sciences	China	179	6700	37.43
8	Sun Yat Sen University	China	163	6244	38.31
9	Nanjing Medical University	China	162	6876	42.44
10	UTMD Anderson Cancer Center	USA	149	13,002	87.26

### Journal influence and citation dynamics in autophagy research

**Fig. 5:**
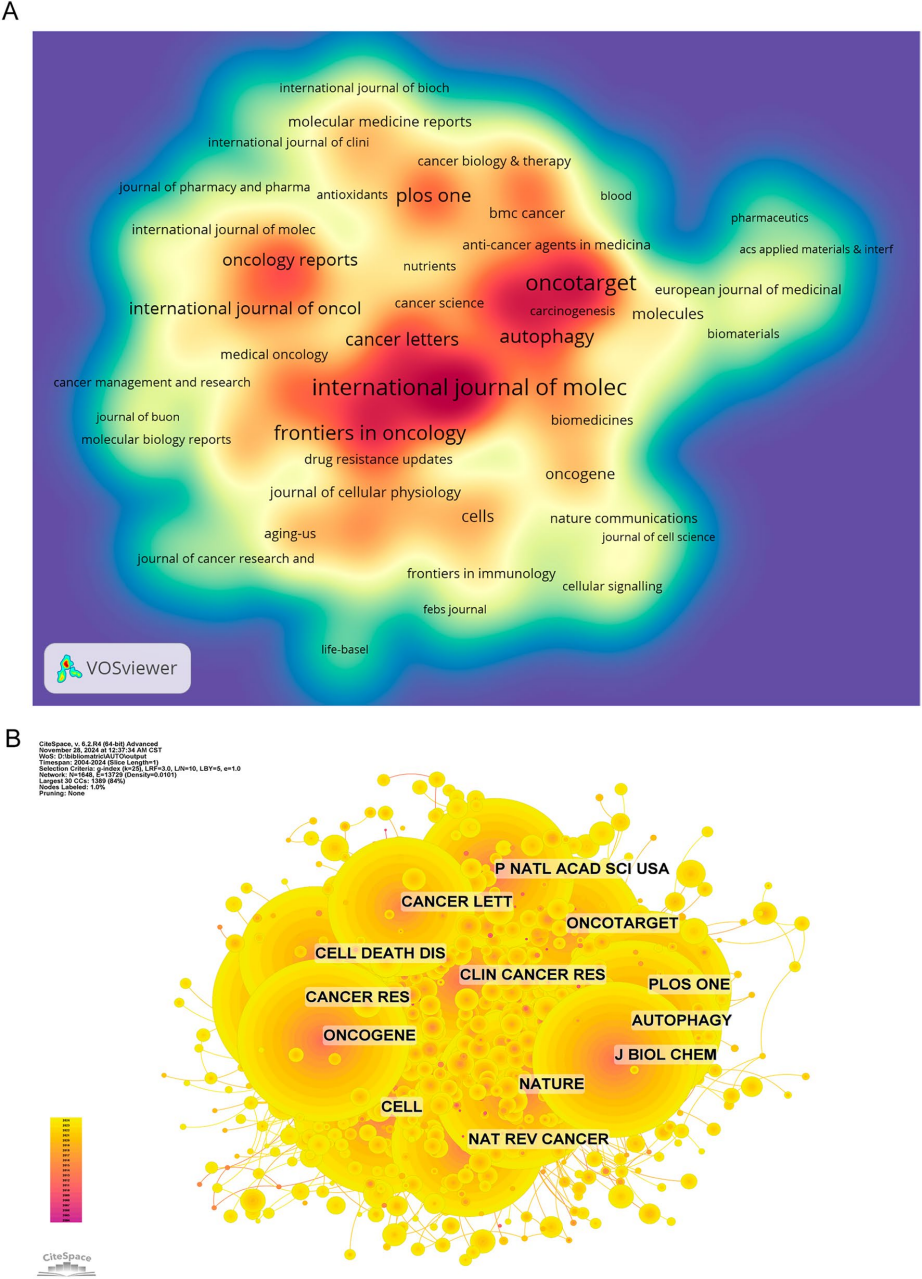
**A** Density map illustrating the concentration of journal publications over time or across different categories. **B** Co-citation network map of journals, showing the relationships between journals based on the frequency of co-citations, highlighting the most influential journals in the field

**Fig. 6:**
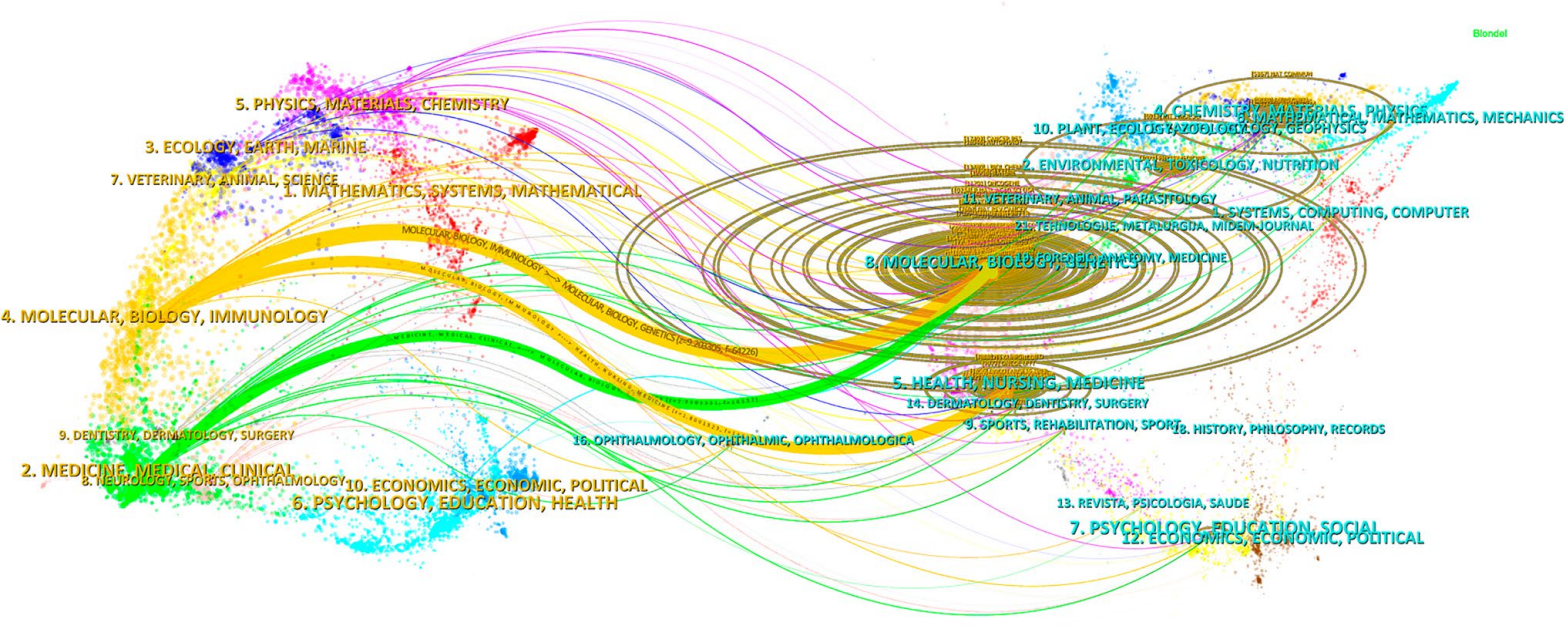
Dual map of journals, displaying the connections between journals in two distinct clusters, often representing different research areas or disciplines within the field of study

The field’s top ten journals, according to publication volume and citation count, are shown in Tables 3 and 4. Out of all the journals that produce research articles, the most prolific one is International Journal of Molecular Sciences with 292 articles (3.15%), followed by Oncotarget with 258 articles (2.78%), Cell Death & Disease with 245 articles (2.64%), and Cancers with 242 articles (2.61%). Autophagy has the greatest impact factor (14.6) among the top ten most prolific journals. The majority of these journals, 90% to be exact, fall into the Q1 and Q2 categories (Fig. 5A). A journal’s impact on the scientific community is reflected in the frequency of its co-citations, which indicate the journal’s influence. Cancer Research has 6118 co-citations, the highest of any journal, according to Fig. 5B and Table 4. Autophagy comes in second with 5582 co-citations, and Cell comes in third with 5393 co-citations. With 4263 citations and the greatest impact factor (72.5), Nature Reviews Cancer stands out among the top 10 most co-cited publications. It is worth mentioning that all of the journals that have been referenced are either Q1 or Q2, which indicates how prominent they are in the subject. The frequency of a journal’s co-citations is a measure of its impact, which reflects its prominence within the scientific community. Table 4 and Fig. 5B show that out of all the journals with at least five thousand citations, Cancer Research has the highest (6118), followed by Autophagy with 5582 citations, and Cell with 5393 citations. With an impact factor of 72.5 and 4263 co-citations, Nature Reviews Cancer is the most cited journal among the top ten. It is worth mentioning that all the journals that are referenced together fall into either the Q1 or Q2 category, which indicated how influential and prominent they are in the subject. An overlay of dual-map analysis is used to depict the topic distribution of academic papers (Fig. 6). The citation connections were depicted by the colored trajectories, which show the journals that cite one another on one side and the journals that are cited on the other. Journals in the molecular/biology/immunology domain cite research from the molecular/biology/genetics domain more often than journals in the medicine/medical/clinical domain, and journals in the health/nursing/medicine domain cite research from the molecular/biology/genetics domain more often than journals in the immunology domain.

**Table 3:** Table of journal publications

Rank	Journal	Article counts	Percentage (9284)	IF	Quartile in category
1	International Journal of Molecular Sciences	292	3.15	4.9	Q1
2	Oncotarget	258	2.78	–	–
3	Cell Death & Disease	245	2.64	8.1	Q1
4	Cancers	242	2.61	4.5	Q1
5	Frontiers in Oncology	209	2.25	3.5	Q2
6	Autophagy	162	1.74	14.6	Q1
7	PLoS One	156	1.68	2.8	Q1
8	Cancer Letters	154	1.66	9.1	Q1
9	Frontiers in Pharmacology	127	1.37	4.4	Q1
10	Biomedicine & Pharmacotherapy	125	1.35	6.9	Q1

**Table 4:** Co-citation table of journals

Rank	Cited Journal	Co-citation	IF (2023)	Quartile in category
1	Cancer Res	6118	12.5	Q1
2	Autophagy	5582	14.6	Q1
3	Cell	5393	45.5	Q1
4	Oncogene	5145	6.9	Q1
5	J Biol Chem	4999	4.0	Q2
6	Nature	4944	50.5	Q1
7	PLoS One	4744	2.8	Q1
8	P Natl Acad Sci USA	4634	9.4	Q1
9	Clin Cancer Res	4334	10.0	Q1
10	Nat Rev Cancer	4263	72.5	Q1

### Analysis of prolific and highly cited authors

**Fig. 7:**
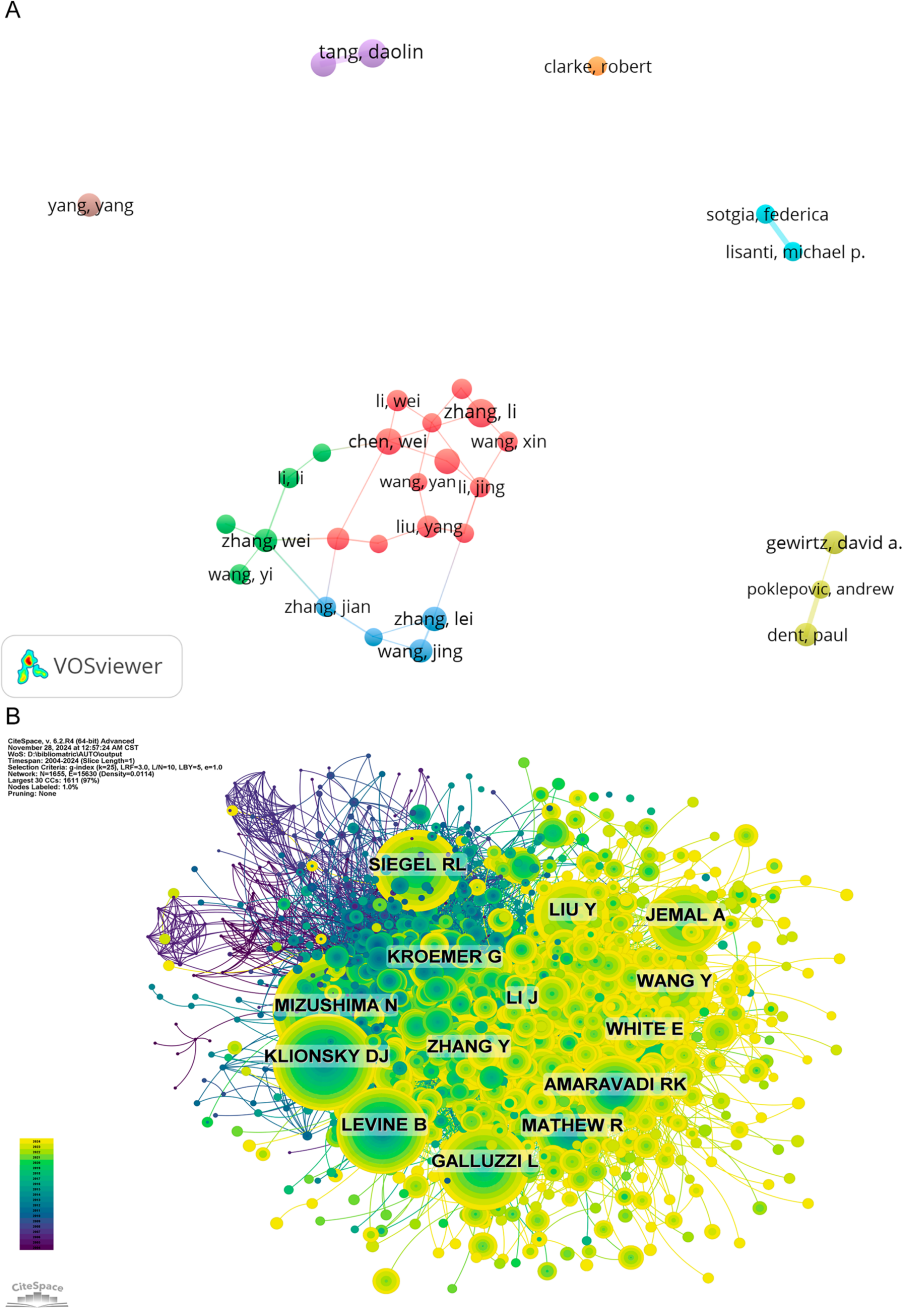
**A** Cooperation network of authors, illustrating the collaborative relationships between researchers based on co-authorship of papers. **B** Co-citation network of authors, depicting the relationships between researchers based on the frequency of co-citations of their works, identifying influential figures in the research community

Table 5 presented an overview of the authors who have contributed most significantly to the literature on autophagy and its role in tumor treatment resistance. Collectively, these authors were responsible for 310 articles, accounting for 3.34% of the total publications in this field. In terms of total publications, Zhang Li led with 36 articles, followed by Tang Daolin with 35, Chen Wei with 32, and Kang Rui with 31. Figure 7A illustrated the CiteSpace visualization of the authors’ collaborative network. Both Table 5 and Fig. 7B highlighted the top ten authors based on citation and co-citation counts. The work of 311 authors was highly regarded, as evidenced by their citation counts exceeding 100. Among the most cited were Klionsky DJ with 1806 citations, followed by Zhang Li with 1412 citations, and Chen Wei with 1164 citations. Notably, these authors were linked to many co-cited works, with Mizushima N being one of the prominent contributors.

**Table 5:** Author’s publications and co-citation table

Rank	Author	Count	Rank	Co-cited author	Citation
1	Zhang, Li	36	1	Mizushima n	1806
2	Tang, Daolin	35	2	Klionsky DJ	1412
3	Chen, Wei	32	3	Levine B	1164
4	Kang, Rui	31	4	Galluzzi L	1039
5	Wang, Wei	31	5	White E	1007
6	Zhang, Lei	30	6	Li J	877
7	Gewirtz, David A	29	7	Zhang Y	864
8	Wang, Jing	29	8	Siegel RL	863
9	Zhang, Wei	29	9	Wang Y	816
10	Dent, Paul	28	10	Amaravadi RK	763

### Analysis of co-cited references

**Fig. 8:**
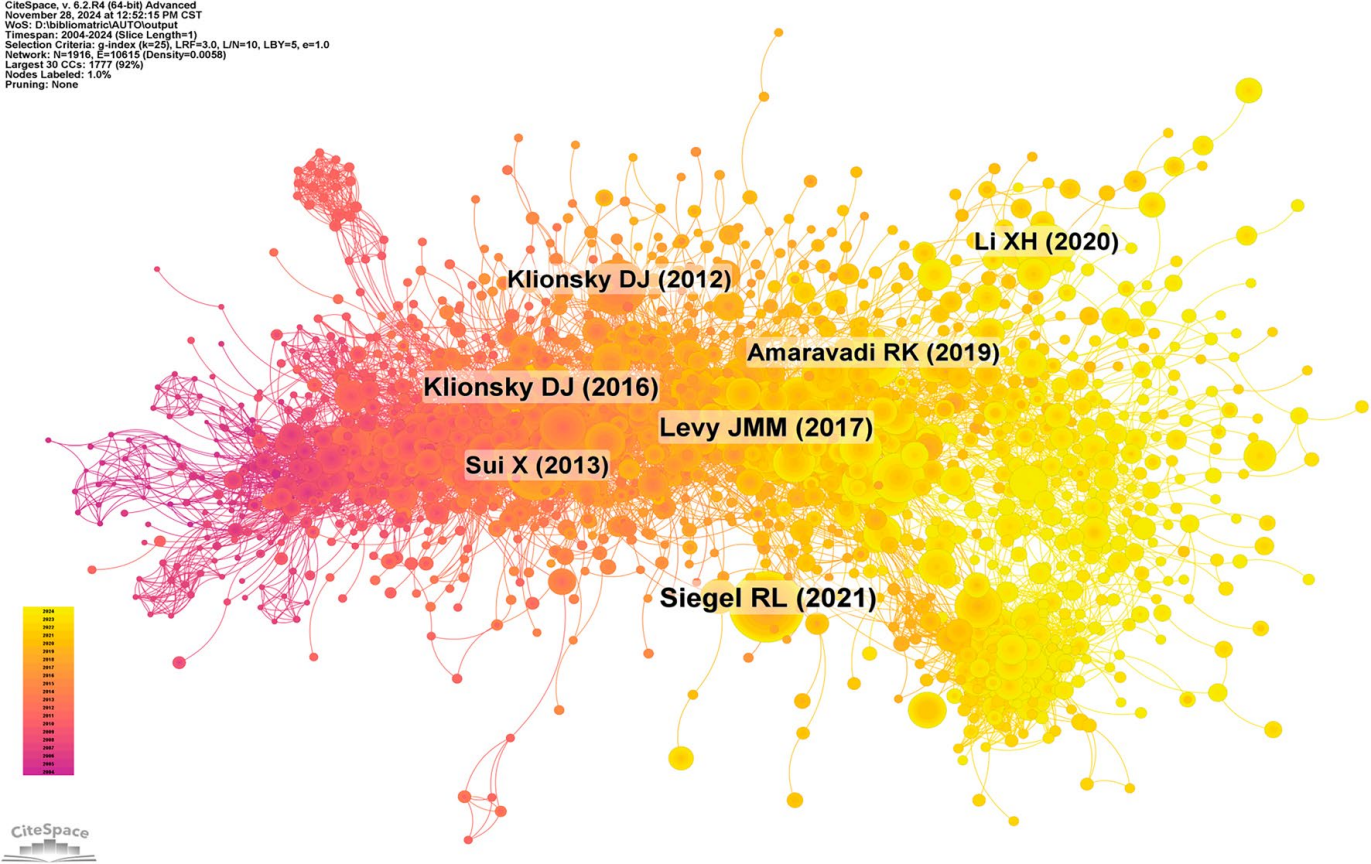
Co-cited network of literature, highlighting the most frequently co-cited articles, showing the interconnections between influential papers in the field

**Fig. 9:**
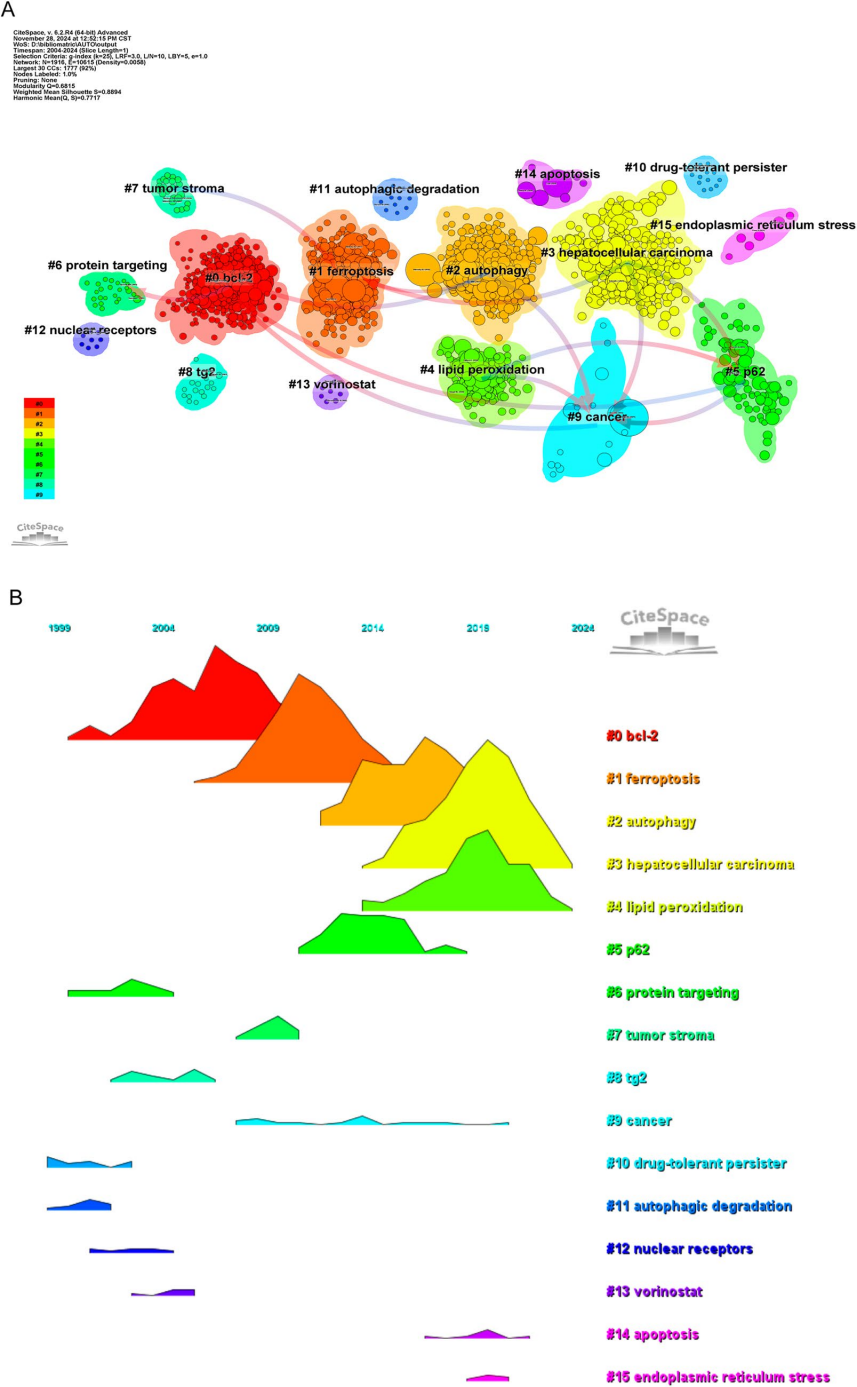
**A** Clustering of co-cited literature, grouping articles based on shared citations to identify emerging trends or themes within the literature. **B** Peak map of co-cited literature, identifying articles with significant spikes in co-citation frequency, indicating influential studies or research topics

There were 1916 nodes and 10,615 linkages in the reference network that will be examined annually from 2004 to 2024 (Fig. 8). Top ten most-cited papers (Table 6) included one from Nature Reviews Cancer, “Targeting autophagy in cancer,” which describes autophagy as a process that sends damaged cell components to lysosomes for destruction. It supplies energy and macromolecular precursors and is essential for cellular component turnover. Interventions that either promote or suppress autophagy have been proposed as cancer treatments due to the fact that autophagy’s function in cancer is context dependent. Because of this, cancer autophagy treatment is a contentious subject. Our goal in writing this review was to help readers understand the context-dependent significance of autophagy and how to use contemporary clinical trial design methodologies to effectively target autophagy treatments. The second-ranked piece is Dieter Riemann’s “Guidelines for the use and interpretation of assays for monitoring autophagy” from Autophagy. In 2008, the first standardized criteria for autophagy research were published, as highlighted in the article. Many more researchers have joined the field since then, and research has progressed at a rapid pace. Our understanding of the world and the technology that surround it is ever growing. Given the importance of autophagy in diverse species, it is imperative that these rules be updated. For this goal, the article discusses a number of different approaches. Still, questions over what counts as an appropriate autophagy assessment method persist, especially when it comes to multicellular eukaryotic organisms. It is important to note that measuring the flow of the autophagy pathway (the entire process) is different from counting or analyzing the volume of autophagic components (such as autolysosomes or autophagosomes) at any point in the autophagic process. Autophagy may be either blocked, leading to an accumulation of autophagosomes, or stimulated, improving transport to lysosomes or vacuoles for destruction in plants and fungi, respectively. The former is more common in higher eukaryotic organisms and some protists, such as Dictyostelium. The idea that more autophagosomes do not always mean more autophagy is crucial to keep in mind, especially for researchers who are just starting out in the area. Even if autophagosome biosynthesis has not altered, blockage of transport to the lysosome is a common cause of autophagosome buildup. There may be less degradation activity if autolysosomes are more numerous. Researchers interested in autophagy and associated processes, as well as reviewers tasked with offering constructive criticism on publications covering these topics, will find this article’s collection of recommendations for choosing and analyzing autophagy assays to be helpful. Because the right detection methods are context and question dependent, these recommendations are not a set of hard and fast rules. Furthermore, it is highly recommended to use numerous approaches to monitor autophagy, as no one method is guaranteed to be suitable in all instances. This manual takes into account the several ways autophagy may be assessed and the data that can or cannot be derived from them. The ultimate goal of this discussion is to promote technical advancement in the field of autophagy detection by outlining the benefits and drawbacks of various techniques. We analyzed the references using temporal clustering and co-citation (Fig. 9A, B). Clusters 0, protein targeting, TG2, drug-tolerant persisters, autophagic degradation, nuclear receptors, and Bcl-2 were identified by our analysis as early research hotspots. Cluster 1, ferroptosis, cluster 5, tumor stroma, and vorinostat are some of the most active areas of study in the medium to long term. Clusters 2–15 contain current and future study subjects in the discipline, including autophagy, hepatocellular carcinoma, lipid peroxidation, cancer, apoptosis, and endoplasmic reticulum stress.

**Table 6:** Co-citation table of literature

Rank	Title	Journal	author(s)	Total citations
1	Targeting autophagy in cancer	Nature Reviews Cancer	Levy JMM	338
2	Guidelines for the use and interpretation of assays for monitoring autophagy	Autophagy	Klionsky DJ	200
3	Targeting autophagy in cancer: recent advances and future directions	Cancer Discovery	Amaravadi RK	192
4	Autophagy and chemotherapy resistance: a promising therapeutic target for cancer treatment	Cell Death & Disease	Sui X	189
5	Autophagy and autophagy-related proteins in cancer	Molecular Cancer	Li XH	187
6	Biological functions of autophagy genes: a disease perspective	Cell	Levine B	162
7	Chloroquine inhibits autophagic flux by decreasing autophagosome-lysosome fusion	Autophagy	Mauthe M	155
8	Mechanism and medical implications of mammalian autophagy	Nature Reviews Molecular Cell Biology	Dikic I	153
9	Deconvoluting the context-dependent role for autophagy in cancer	Nature Reviews Cancer	White E	143
10	Autophagy and multidrug resistance in cancer	Chinese Journal of Cancer	Li YJ	143

### Keyword analysis and research hotspots

**Fig. 10:**
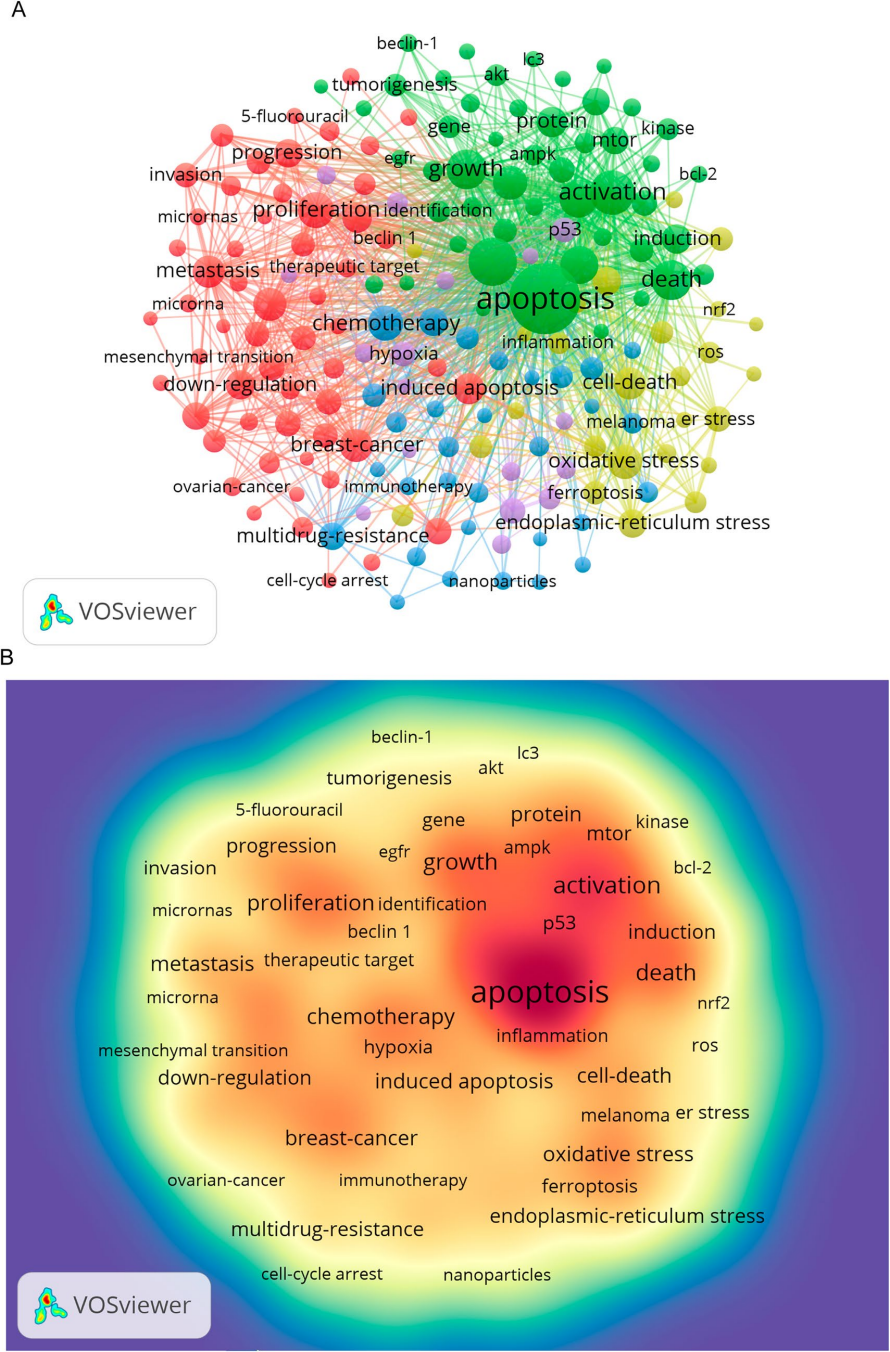
**A** Network map of high-frequency keywords, visualizing the relationships between commonly used terms in the literature, highlighting key areas of focus in the research field. **B** Density map of keywords, showing the concentration of specific keywords across publications, providing insight into the most researched topics

**Fig. 11:**
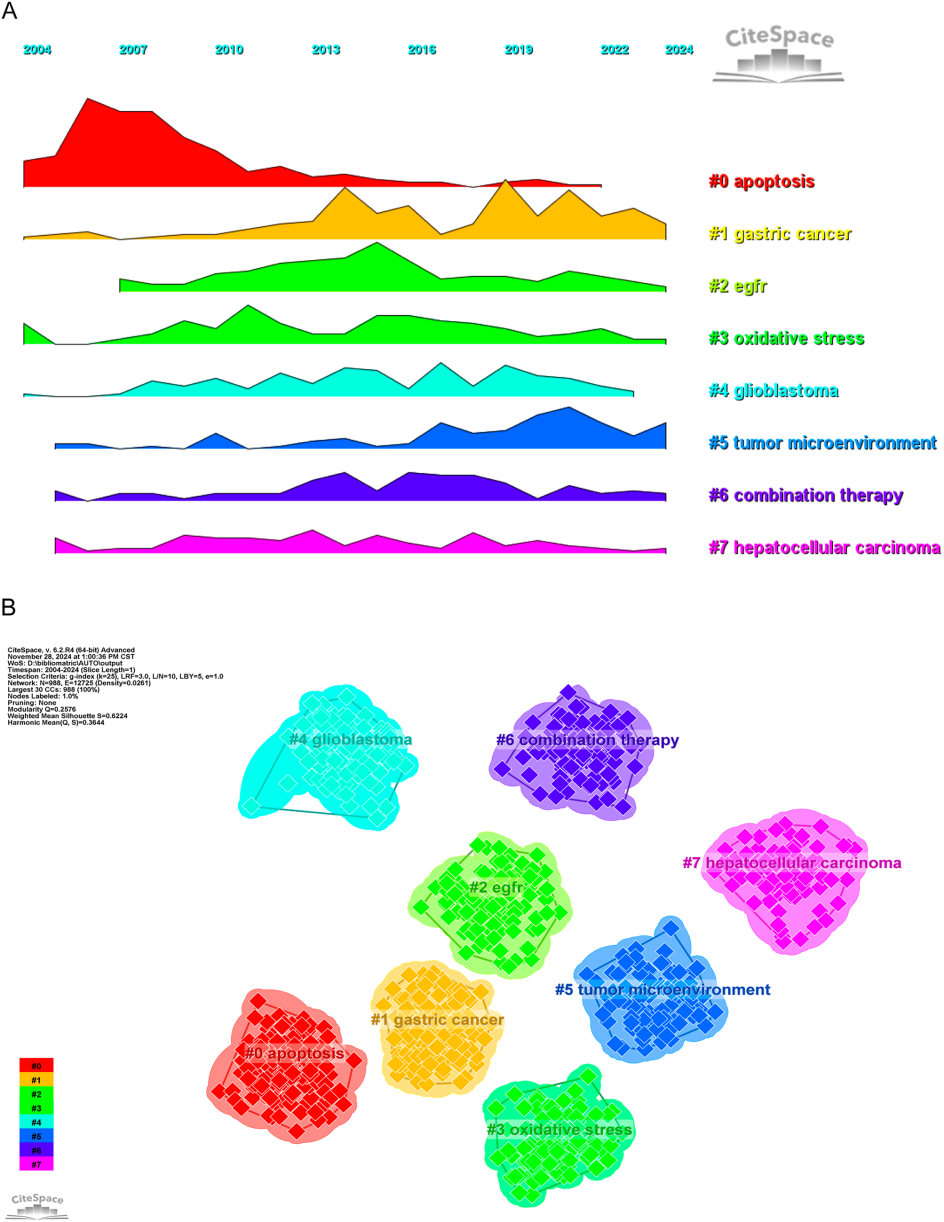
**A** Peak map of keyword clustering, identifying periods or specific areas of intense research activity based on keyword frequency and clustering. **B** Clustering map of keywords, grouping related terms to reveal thematic clusters within the literature

The current status and future prospects of a field can be rapidly illuminated by examining its term usage. The following terms appear often in VOSviewer data: “apoptosis” (3179 instances), “inhibition” (1330 occurrences), “activation” (1083), “growth” (850 occurrences), and “death” (826 occurrences) (Table 7; Fig. 10A, B). After removing any unnecessary terms, we built a network using 180 keywords that occurred at least 84 times, which led to the formation of five separate clusters. Cluster 1 (red) includes 56 keywords, such as “proliferation,” “chemoresistance,” “signaling pathway,” “breast cancer,” “colorectal cancer,” “NF kappa B,” “microRNAs,” “invasion,” “migration,” “prognosis,” “induced apoptosis,” “angiogenesis,” “cancer therapy,” “metastasis,” “poor prognosis,” “promotes,” “stem cells,” and “up regulator.” Cluster 2 (green) contains 37 keywords, including “apoptosis,” “death,” “inhibition,” “survival,” “activation,” “protein,” “rapamycin,” “receptor,” “macroautophagy,” “kinase,” “induction,” “AKT,” “complex,” “degradation,” “EGFR,” “differentiation,” “modulation,” “PI3K,” “protein,” “suppression,” and “target.” Cluster 3 (blue) consists of 31 keywords, such as “antitumor activity,” “chloroquine,” “cycle arrest,” “cytotoxicity,” “delivery,” “drug,” “efficacy,” “immunotherapy,” “combination therapy,” “nanoparticle,” “sorafenib,” and “doxorubicin.” Cluster 4 (yellow) includes 27 keywords, such as “cell death,” “ER stress,” “oxidative stress,” “ferroptosis,” “ROS,” “NRF2,” “inflammation,” “activated protein kinase,” “glycolysis,” “metabolism,” “metformin,” “mitochondria,” “necrosis,” “ROS,” and “reactive oxygen species.” Cluster 5 (purple) contains 17 keywords, including “glioma,” “cancer stem cell,” “microenvironment,” “p53,” “radiation,” “senescence,” “sensitivity,” and “hypoxia.” We created a volcano plot using CiteSpace to visually display how research hotspots have changed over time (Fig. 11A, B). Our analysis reveals that current research hotspots include “apoptosis,” “gastric cancer,” “EGFR,” “oxidative stress,” “glioblastoma,” “tumor microenvironment,” “combination therapy,” and “hepatocellular carcinoma.”

**Table 7:** High frequency keyword table

Rank	Keyword	Counts	Rank	Keyword	Counts
1	Apoptosis	3179	11	Oxidative stress	582
2	Inhibition	1330	12	Breast-cancer	569
3	Activation	1083	13	Chemoresistance	569
4	Growth	850	14	Metastasis	533
5	Death	826	15	Cell-death	531
6	Mechanisms	761	16	Induced apoptosis	493
7	Pathway	760	17	Protein	467
8	Proliferation	691	18	Induction	437
9	Chemotherapy	648	19	Cisplatin	428
10	Survival	584	20	Down-regulation	428

### Co-cited references and keywords

**Fig. 12:**
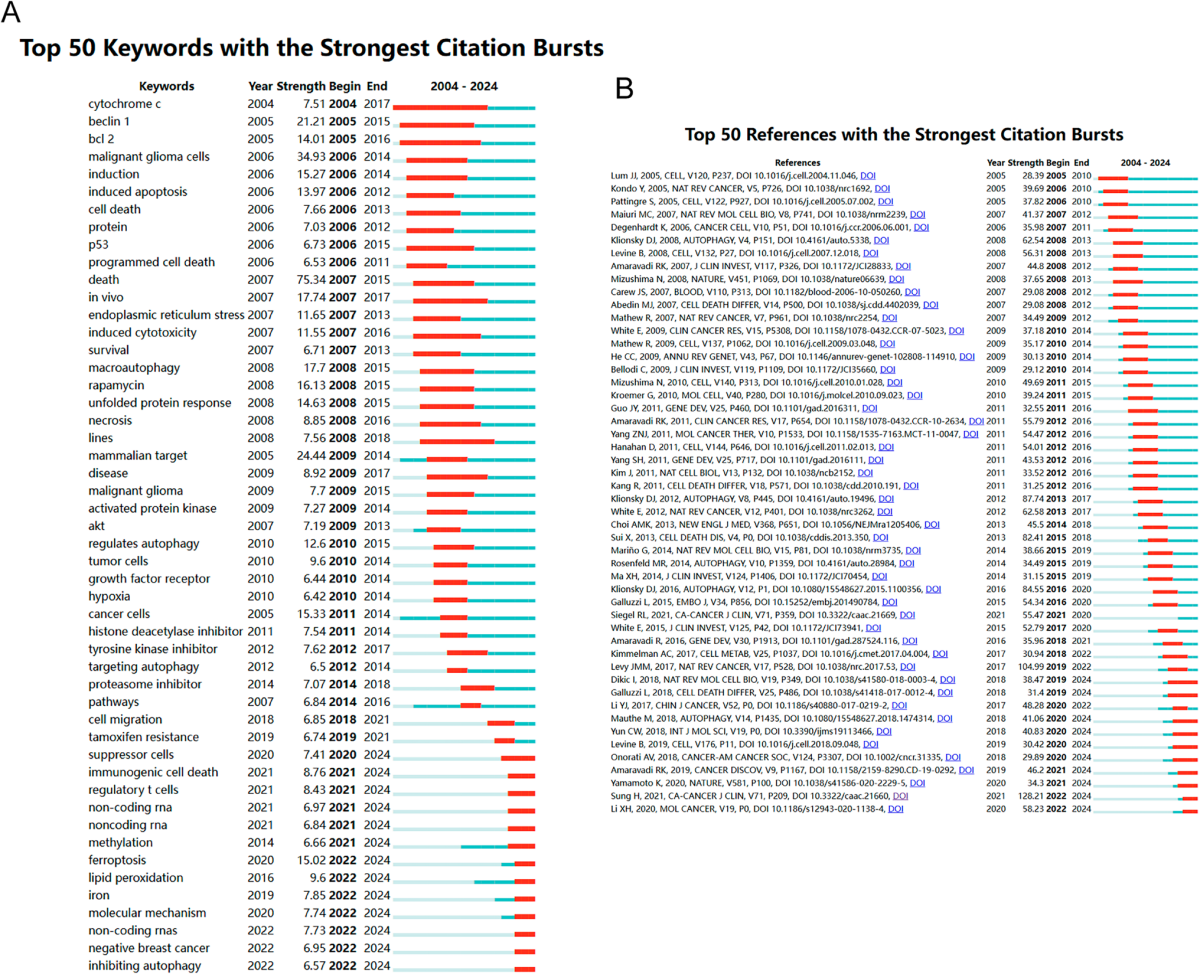
**A** Bursting map of cited literature, visualizing articles that have experienced significant spikes in citations, indicating groundbreaking or highly influential work. **B** Bursting map of keywords, highlighting keywords that have seen a surge in usage over a specific period, reflecting emerging research trends or topics

Using CiteSpace, we identified the 50 most significant citation bursts in the field of autophagy and drug resistance in tumors. The article ‘Targeting Autophagy in Cancer,’ published in Nature Reviews Cancer and authored by Jean M. Mulcahy Levy, emerged as the most cited publication after excluding extraneous references. This research explores autophagy, a cellular mechanism that enables the recycling of cellular components by transporting them to the lysosome for degradation. This process not only generates energy but also produces macromolecular precursors. Given the complexity and context-dependent nature of autophagy’s role in cancer, interventions that either promote or inhibit autophagy have been proposed as potential cancer therapies. Consequently, the search for cancer treatments targeting autophagy has been the subject of considerable debate. The review emphasizes the importance of understanding autophagy’s environmental dependence and the need for effective targeted strategies through contemporary clinical trial design methodologies. Notably, all 50 articles included in our analysis were published between 2004 and 2024, reflecting their high citation impact over the past two decades. The significance of autophagy in tumor drug resistance is likely to remain a focal point of future research, as ten of these studies are currently experiencing substantial citation activity (Fig. 12A). We also identified the 50 most prevalent terms from the 756 citation bursts in this field, which are depicted in Fig. 12B. These keywords represent the core areas of active research and highlight potential directions for further exploration.

## Discussion

### Trend of autophagy and drug resistance research

The findings from the bibliometric analysis on autophagy and tumor drug resistance provide significant insights into the evolution of this research field and its current trajectory [38]. The steadily increasing number of publications since 2004, especially the rapid surge after 2019, reflects the growing recognition of autophagy as a crucial mechanism in cancer biology and drug resistance. This uptrend aligns with advances in research technologies, increased funding, and a broader understanding of the role autophagy plays in mediating therapeutic resistance in tumors. The peak in 2021 highlights a pivotal moment in the field, signaling a shift towards more concentrated efforts to target autophagy for overcoming cancer therapy resistance. The global nature of this research, spanning 103 countries and regions, and involving thousands of institutions and researchers, emphasizes the widespread and collaborative nature of efforts in this area [39, 40]. China, in particular, stands out not only in publication volume but also in citation count, indicating a leading role in the research community. However, China’s relatively lower citation-to-publication ratio suggests that while the quantity of research output is high, there is still room for improvement in the quality of publications [41]. The United States, with its high citation-to-publication ratio, continues to be a prominent player in the field, reflecting a well-established academic infrastructure that produces high-impact research. The collaboration patterns further underscore the importance of international partnerships in advancing the understanding of autophagy in cancer. China’s collaboration with countries such as South Korea, Japan, and Iran highlights the region’s strong focus on this field, while the United States’ network with Western countries suggests established academic ecosystems in cancer research.

The dominance of Chinese institutions in the top 10 research institutions further highlights the country’s significant investment and leadership in the field. The University of Texas System, with its high output and citation impact, underscores the ongoing strength of research in the United States. However, the finding that domestic institutions tend to collaborate predominantly within their own countries points to a potential barrier to broader international collaboration. Stronger cross-border cooperation could foster the exchange of ideas and enhance the global synergy necessary to tackle the complex challenges posed by tumor drug resistance. The influence of leading journals such as Cancer Research, Autophagy, and Cell in the field demonstrates the centrality of these publications in shaping research directions. The high impact factor and co-citation frequency of these journals indicate their significant role in advancing the field’s understanding of the intricate relationship between autophagy and cancer drug resistance. The Q1 and Q2 categorization of most of the top journals highlights the importance of publishing in high-quality, well-regarded outlets to gain visibility and recognition.

The thematic analysis using dual-map overlay analysis provides a clear picture of the evolving research landscape. Early research hotspots focused on the fundamental mechanisms of autophagy and its role in cancer cell survival, with a particular focus on protein targeting and autophagic degradation [28, 42, 43]. As the field has matured, newer research trends have emerged, such as the study of ferroptosis, lipid peroxidation, and autophagy’s role in tumor microenvironments. Current research clusters emphasize not only autophagy but also its intersections with other critical processes like hepatocellular carcinoma, endoplasmic reticulum stress, and apoptosis [44–46]. The progression of research topics from basic mechanisms to more complex interactions reflects an expanding understanding of how autophagy modulates tumorigenesis and therapeutic resistance. Emerging topics such as lipid peroxidation and autophagy’s impact on hepatocellular carcinoma signify a shift towards exploring how autophagy may influence other cellular processes and cancer types beyond the traditional focus on apoptosis and necrosis.

### Research hotspots

The complex role of autophagy in cancer progression and therapy resistance has generated considerable interest, with multiple studies focusing on either stimulating or inhibiting autophagy for therapeutic purposes. Future research will likely delve deeper into the environmental and tumor-specific context of autophagy, examining how different types of tumors might require distinct strategies. Furthermore, as targeted therapies for autophagy modulation advance, understanding the balance between its pro-survival and anti-survival roles will be crucial.

The keyword “combination therapy” emerges as a significant area of interest, indicating that researchers are increasingly considering how autophagy inhibition or activation can complement other therapeutic modalities [47, 48]. This could include chemotherapy, immunotherapy, or targeted therapies. Research in this area will likely explore synergistic effects of combining autophagy modulators with existing cancer treatments, aiming to overcome drug resistance and enhance treatment efficacy.

The connection between oxidative stress and autophagy (e.g., “oxidative stress,” “ROS,” “NRF2”) is another prominent research area [49, 50]. The role of reactive oxygen species (ROS) in autophagic regulation and tumor progression is a complex one, with ROS potentially serving as both inducers of autophagy and as contributors to cellular damage [51, 52]. Future studies may investigate how manipulating ROS levels can be leveraged to control autophagy in cancer cells and improve treatment outcomes.

Keywords such as “tumor microenvironment,” “glioma,” and “cancer stem cells” indicate an increasing focus on the non-cancerous components surrounding tumors [53–55]. Understanding how autophagy interacts with the immune system, stromal cells, and other elements of the tumor microenvironment could open new avenues for targeting autophagy to enhance the effectiveness of cancer therapies. This research might focus on how autophagy in the microenvironment influences cancer cell survival and metastasis.

Several cancers such as gastric cancer, glioblastoma, and hepatocellular carcinoma are highlighted as important areas of study. Future research may focus on understanding how autophagy contributes to drug resistance in these specific cancers, identifying novel biomarkers or therapeutic targets to improve treatment outcomes. For example, researchers might explore how autophagy modulates drug efflux pumps, drug metabolism, or the repair of chemotherapy-induced damage in these tumor types.

The integration of autophagy with immunotherapy is a rapidly emerging field. Keywords like “immunotherapy” and “combination therapy” suggest that autophagy could influence the immune response in cancer treatment. Research might focus on understanding how autophagy affects tumor immune evasion and immune cell function, potentially leading to strategies that enhance the effectiveness of immunotherapies.

As the field moves towards precision medicine, there will likely be a push to tailor autophagy-targeting therapies to specific tumor types based on their molecular and genetic profiles. Studies may investigate the molecular mechanisms underlying autophagy regulation in various cancer subtypes and how this relates to drug resistance patterns, ultimately enabling more personalized and effective treatments.

### Implications for future research

The current state of autophagy and drug resistance research reveals both significant progress and areas that require further investigation. The field has matured from foundational studies to complex analyses of autophagy’s role in various aspects of cancer biology. However, there remain unanswered questions, particularly regarding the context-dependent effects of autophagy. While autophagy can act as a tumor suppressor under certain conditions, it may also promote cancer progression and drug resistance in other contexts. Understanding these dual roles and developing strategies to modulate autophagy for therapeutic purposes remain major challenges. The identification of new biomarkers and the development of targeted therapies to modulate autophagy will be critical in overcoming drug resistance in cancer. Future research should continue to focus on elucidating the molecular pathways that regulate autophagy and its crosstalk with other cellular processes such as apoptosis, lipid metabolism, and oxidative stress. Additionally, there is a need for more comprehensive clinical trials that explore the therapeutic potential of autophagy modulation in cancer treatment. Moreover, International collaboration holds significant implications for the future of autophagy-targeted therapies in several ways. First, cross-border cooperation brings together expertise from various fields, promoting the global exchange of knowledge and ideas, which accelerates the discovery of new autophagy-related biomarkers and therapeutic targets. By pooling global expertise, international collaboration can quickly drive the development of new therapeutic strategies and adapt them to the needs of different patient populations. Second, international collaboration enables clinical trials to include diverse patient populations from various ethnic backgrounds and geographical locations. This helps understand the efficacy of autophagy-targeted therapies across different genetic backgrounds and environmental exposures, promoting personalized treatment approaches. Additionally, international cooperation can integrate resources, funding, and clinical trial sites, which accelerates drug development and clinical testing, reducing the time for therapies to reach the market and improving the reliability of trial results. Furthermore, international collaboration attracts more financial support from global funding agencies, providing essential resources for autophagy-targeted therapy research. Finally, through global cooperation, researchers can standardize treatment protocols, ensuring consistency in the application of therapies across different healthcare settings. In summary, international collaboration will help overcome the challenges faced by autophagy-targeted therapies, drive the clinical translation of therapeutic strategies, and ensure that these therapies benefit diverse patient populations worldwide. In conclusion, the growing body of research on autophagy and tumor drug resistance highlights the importance of this field in cancer biology. The rapid increase in publications, coupled with the global collaboration network, underscores the significance of autophagy in overcoming therapeutic resistance. Future research efforts should focus on translating these findings into clinical practice by developing strategies to target autophagy in a more context-specific manner, paving the way for novel cancer therapies.

## Limitation

This study has several limitations. Firstly, this analysis typically relies on specific databases such as Web of Science, PubMed, and Scopus. These databases may not cover all fields of literature, particularly non-English publications, grey literature (such as conference papers, reports, etc.), or high-quality research published in niche databases. This may lead to an incomplete assessment of the research field. Secondly, citations take time to accumulate, especially for new high-quality literature, which may not be promptly reflected in the analysis results. This can lead to an underestimation of recent research findings, affecting the accurate assessment of trends and hotspot areas. Thirdly, since bibliometric analysis relies on the data quality within databases, errors or incompleteness in the data (such as missing or incorrect citation information, inaccurate journal details, etc.) may affect the reliability of the analysis results. Fourthly, although citation counts are an important metric for assessing the influence of literature, they do not fully reflect the quality or innovation of the research. Additionally, studies in certain fields may not receive high citations in the short term. Finally, Bibliometric analysis reveals research trends but cannot delve into the causal relationships or experimental details within the studies. Therefore, it provides a macro view of the field rather than in-depth insights into specific research mechanisms.

## Conclusion

Research on autophagy and tumor drug resistance in the WoSCC database includes a total of 9284 papers from 103 countries and regions, with the majority concentrated in China, the United States, Italy, South Korea, and Germany. Since 2004, the number of publications has steadily increased, particularly after 2019, reflecting the growing importance of autophagy in tumor drug resistance research. China has contributed nearly 50% of the research, though its citation rate is relatively low, while the United States has a higher citation rate. In terms of research institutions, China has the largest number of institutions, with strong domestic collaborations, though international cooperation could be further strengthened. Regarding journals, International Journal of Molecular Sciences has the highest publication output, and Autophagy has the highest impact factor, with most relevant journals being classified as Q1 or Q2, demonstrating high recognition of autophagy research. Prominent authors such as Mizushima N, Klionsky DJ, and Levine B have significant academic influence. Citation analysis shows that the most referenced papers are Targeting autophagy in cancer and Guidelines for the use and interpretation of assays for monitoring autophagy. Research hotspots include Bcl-2, drug resistance, ferroptosis, and others. Keyword analysis reveals that the research mainly focuses on tumor chemotherapy resistance, apoptosis, oxidative stress, and ferroptosis.

## Data Availability

The datasets generated during and/or analyzed during the current study are available from the corresponding author upon reasonable request.
